# Deep autoencoder-powered pattern identification of sleep disturbance using multi-site cross-sectional survey data

**DOI:** 10.3389/fmed.2022.950327

**Published:** 2022-07-29

**Authors:** Hyeonhoon Lee, Yujin Choi, Byunwoo Son, Jinwoong Lim, Seunghoon Lee, Jung Won Kang, Kun Hyung Kim, Eun Jung Kim, Changsop Yang, Jae-Dong Lee

**Affiliations:** ^1^Department of Clinical Korean Medicine, Graduate School, Kyung Hee University, Seoul, South Korea; ^2^KM Science Research Division, Korea Institute of Oriental Medicine, Daejeon, South Korea; ^3^Department of Korean Medicine, Combined Dispensary, 7th Corps, Republic of Korea Army, Icheon-si, South Korea; ^4^Department of Acupuncture and Moxibustion, Wonkwang University Gwangju Korean Medicine Hospital, Gwangju, South Korea; ^5^Department of Acupuncture and Moxibustion, College of Korean Medicine, Kyung Hee University, Seoul, South Korea; ^6^School of Korean Medicine, Pusan National University, Yangsan, South Korea; ^7^Department of Acupuncture and Moxibustion Medicine, Dongguk University Bundang Oriental Hospital, Seongnam-si, South Korea

**Keywords:** deep autoencoder, deep learning, pattern identification, clustering, sleep

## Abstract

Pattern identification (PI) is a diagnostic method used in Traditional East Asian medicine (TEAM) to select appropriate and personalized acupuncture points and herbal medicines for individual patients. Developing a reproducible PI model using clinical information is important as it would reflect the actual clinical setting and improve the effectiveness of TEAM treatment. In this paper, we suggest a novel deep learning-based PI model with feature extraction using a deep autoencoder and *k*-means clustering through a cross-sectional study of sleep disturbance patient data. The data were obtained from an anonymous electronic survey in the Republic of Korea Army (ROKA) members from August 16, 2021, to September 20, 2021. The survey instrument consisted of six sections: demographics, medical history, military duty, sleep-related assessments (Pittsburgh sleep quality index (PSQI), Berlin questionnaire, and sleeping environment), diet/nutrition-related assessments [dietary habit survey questionnaire and nutrition quotient (NQ)], and gastrointestinal-related assessments [gastrointestinal symptom rating scale (GSRS) and Bristol stool scale]. Principal component analysis (PCA) and a deep autoencoder were used to extract features, which were then clustered using the *k*-means clustering method. The Calinski-Harabasz index, silhouette coefficient, and within-cluster sum of squares were used for internal cluster validation and the final PSQI, Berlin questionnaire, GSRS, and NQ scores were used for external cluster validation. One-way analysis of variance followed by the Tukey test and chi-squared test were used for between-cluster comparisons. Among 4,869 survey responders, 2,579 patients with sleep disturbances were obtained after filtering using a PSQI score of >5. When comparing clustering performance using raw data and extracted features by PCA and the deep autoencoder, the best feature extraction method for clustering was the deep autoencoder (16 nodes for the first and third hidden layers, and two nodes for the second hidden layer). Our model could cluster three different PI types because the optimal number of clusters was determined to be three *via* the elbow method. After external cluster validation, three PI types were differentiated by changes in sleep quality, dietary habits, and concomitant gastrointestinal symptoms. This model may be applied to the development of artificial intelligence-based clinical decision support systems through electronic medical records and clinical trial protocols for evaluating the effectiveness of TEAM treatment.

## Introduction

Pattern identification (PI), a diagnostic method in Traditional East Asian medicine (TEAM), is a meaningful step for TEAM doctors when making treatment decisions such as selection of an appropriate acupuncture point and herbal medicine. It uses clinical information based on traditional diagnostic criteria, which include observation, listening, questioning, and pulse detection (1). Particularly, the use of PI in selecting an optimal combination with a few acupuncture points has been an important research subject to reveal those used in actual clinical practice (2, 3). Most clinical trials on the effectiveness of acupuncture treatment used a fixed-point approach, which is different from the clinical practice that uses a more individualized approach (4). Although some study designs such as conventional randomized clinical trials (RCTs) with a personalized acupuncture protocol or a pragmatic clinical trial have been suggested to overcome the gap between acupuncture research and clinical practice, the results of an individualized approach vs. a fixed-point approach are still controversial (5–9). Nonetheless, several recent experimental studies have supported the significance of acupuncture point selection (10–13). Therefore some studies with data-mining methods were conducted using RCT data, medical records, virtual diagnosis data, and classical medical texts to systematically prove the relationship between symptoms, diseases, PI, and acupuncture point selections (3, 14–16).

Artificial intelligence (AI) techniques have also emerged in the research of TEAM. Previous studies used artificial neural network models to differentiate patterns for acupuncture point selections (17, 18), and clustering algorithms to discover the combination rules of herbal medicine (19). Also, the recent deep learning models such as bidirectional encoder representations from transformers generated some new herbal medicine prescriptions from a few medical records (20, 21). However, to the best of our knowledge, there are few AI studies that assist PI from large amounts of clinical information, though most clinical guidelines recommend a PI process by a TEAM doctor prior to providing acupuncture or herbal medicine treatment (22, 23).

With the appropriate PI, a wide variety of conditions can be addressed by TEAM treatment. Sleep disturbances were one of the major target conditions for TEAM treatment in several previous studies (19, 24–28). The Korean Medicine Clinical Practice Guidelines for insomnia disorder, which was officially developed by research funded by the government, suggest that TEAM doctors may consider six types of PI before TEAM treatment (29). Furthermore, a recent systematic review for insomnia showed that acupuncture treatment using PI significantly improved the total effectiveness rate compared to conventional medication (30). However, the effect of TEAM treatment using PI is not reproducible since PI types and processes may be inconsistent among TEAM doctors in clinical settings. Therefore, the development of a model that can consistently produce the same PI for certain patient details required.

In this paper, we suggested a novel data-driven PI method for TEAM treatment using emerging bioinformatics techniques in combination with feature extraction using a deep autoencoder, one of the self-supervised deep learning models, and clustering using *k*-means clustering, an unsupervised machine learning model. To develop a new model using various types of clinical information as input data and provide reproducible PI as an output for TEAM treatment decisions in patients with sleep disturbances, we used cross-sectional study data which examined the association between sleep and diet/digestion in Republic of Korea Army (ROKA) active duty service members.

## Materials and methods

### Study population

A multi-site cross-sectional study was conducted using an anonymous electronic survey. The study was posted in five units of the ROKA through printed recruitment posters and electronic bulletin boards from August 16, 2021, to September 20, 2021. The participants were recruited during the same period. The original aim of this study was to examine the association between sleep and diet/digestion in ROKA active duty service members. The results will be published in another paper.

Among active duty service members in five units of the ROKA who met the inclusion criteria, the participants who provided informed consent were enrolled in the study. The inclusion criteria were (1) age 19 years or over; (2) active duty service members (private, private first class, corporal, and sergeants) who completed the basic military training course, and (3) those who voluntarily agreed to participate in the study. There were no exclusion criteria.

### Sample size calculation for cross-sectional study

Assuming that the total number of all active duty service members in the ROKA is approximately 300,000, the sample size was calculated using the following equation. The margin of error was 3% and the confidence level was 95%, and the sample size result was 1,064 (the target number of completed surveys).


$$\begin{array}{l} Sample\;size\;=\;\frac{\frac{z^{2}\times p\left(1-p\right)}{e^{2}}}{1+\;(\frac{z^{2}\times p(1-p)}{e^{2}N})}\\ \;(N\;=\;numberinthepopulations\text{;}\\ \;e\;=\;marginoferror;\;z\;=\;Z-score) \end{array}$$

Previous studies using surveys showed that various factors such as the survey method, survey content, and participant compensation were associated with the response rate of the study subjects. In particular, the online survey method is known to have about a 10% lower response rate compared to other media, but the actual response rate was different in each study (31). In this study, referring to the response rate (3.4%) reported in a previous study that conducted a health-related survey in adult males, the response rate was set to 3%, and the target number of questionnaires was determined to be 35,467 (31).

### Survey instrument of cross-sectional study

The survey instruments were refined to reveal the military environment by healthcare professionals (seven TEAM doctors including five military doctors). This involved the refinement of the questionnaire by changing the phrasing and modifying questions to clarify the premise of each item within the questionnaire. The final questionnaire was designed and distributed through the web-based application Survey Monkey.

The survey consisted of six sections: (1) demographics (birth, recruitment date, height, weight, military identification number, rank, military unit, education, smoking status, alcohol consumption habits, caffeine consumption, exercise, and physical grade); (2) medical history (present/past history of sleep disorders, present/past history of gastrointestinal disorders, present/past history of general diseases including hypertension, diabetes, hyperlipidemia, and cardiac disease, stress status, and drug history); (3) military duty (branch, position, night shift with or without tomorrow duty-off, and its effect on sleep and/or fatigue); (4) sleep-related assessments (Pittsburgh sleep quality index (PSQI), Berlin questionnaire, and sleeping environment); (5) diet/nutrition-related assessments [dietary habit survey questionnaire and nutrition (32) quotient (NQ)]; and (6) gastrointestinal-related assessments [gastrointestinal symptom rating scale (GSRS) and Bristol stool scale (BSS)].

The PSQI, a self-assessment questionnaire to evaluate sleep quality within the past month, contains 19 items consisting of seven component scores, including sleep quality, sleep latency, sleep duration, daytime dysfunction, sleep efficiency, sleep disturbances, and sleeping medication use (33). A final score of >5 out of 21 indicates significant sleep disturbance.

The Berlin questionnaire has 11 questions grouped into three categories (34). The first category comprises five questions concerning snoring, witnessed apnea, and the frequency of such events. The second category comprises four questions addressing daytime sleepiness, with a sub-question on drowsy driving. The third category comprises two questions concerning a history of high blood pressure (> 140/90 mmHg) and a body mass index (BMI) of >30 kg/m^2^. Categories 1 and 2 were considered positive if there were two positive responses in each category, while category 3 was considered positive with a self-report of high blood pressure and/or a BMI of > 30 kg/m^2^. The study patients were scored as being at high risk of having obstructive sleep apnea (OSA) if the scores were positive for two or more of the three categories.

The dietary habit survey questionnaire consists of 25 items to evaluate the dietary habits of Korean adults (35). It includes the number of meals per day, mealtime regularity, the amount consumed, time taken for a meal, the frequency of missed meals, the frequency of having breakfast, the reason for missing breakfast, the frequency of dinners with family, the frequency of overeating, meal at which overeating occurred (breakfast, lunch, dinner or not), the frequency of eating out, the frequency of eating snacks, the time of eating snacks, the types of snacks, the time of late-night meals, whether certain foods were not eaten, the reasons for not eating certain foods, and the frequency of food intake (grains, meat, fish, eggs and legumes, fruits, vegetables, milk and dairy products, fatty foods, instant foods, and fast foods).

The NQ comprehensively evaluates the nutritional status and meal quality of individuals or groups of Korean adults through a checklist consisting of 21 items (36). It provides the global NQ score (NQ global), and scores for four factors: nutritional balance (NQ balance), food diversity (NQ diversity), moderation in the amount of food eaten (NQ moderation), and dietary behavior (NQ behavior). It is considered “good” if the score is 58 or higher, and “monitoring is necessary” if it is below 58.

The GSRS evaluates gastrointestinal symptoms *via* an inquiry table consisting of 15 items for the evaluation of general gastrointestinal symptoms (37). Each GSRS item is rated on a 7-point Likert scale ranging from “no discomfort” to “very severe discomfort.”

The BSS examines the stool status in the past 24 h (32). The score is based on a one to seven scale where one corresponds to a hard stool and seven corresponds to watery diarrhea.

###  Data preprocessing

Data preprocessing to improve data quality and impute missing values was performed in three steps. In the first step, from all survey responders, the participants who provided multiple responses were eliminated to ensure survey reliability. Second, the participants who did not meet the inclusion criteria were removed. The participants who completed the survey remained. Last, a few samples with outliers, which might be caused by miswriting in open question items such as height, weight, smoking amount, and smoking duration were also eliminated after exploratory data analysis (EDA).

Each PSQI, Berlin questionnaire, NQ, and GSRS score was calculated and the remaining questionnaire responses were used for input data. In clinical practice, TEAM doctors' questions to patients are closer to each item of the questionnaire, and conversely, calculating each questionnaire's scores one by one is closer to the purpose of the clinical study. The calculated scores were used for external cluster evaluation.

Since this study was conducted to examine patients with sleep disturbances, the participants with PSQI scores of over five were collected as a total data set. Then, the data set was randomly split into a training set (80%) and test set (20%) for evaluating the machine learning models.

### Feature extraction

The autoencoder is a simple unsupervised learning model. It learns hidden features through encoding and decoding unlabeled data. Consider a *d*-dimensional data set *X* = {*x*_1_, *x*_2_, …, *x*_*d*_}, where *d* is the number of variables presented at the input layer. The autoencoder attempts to reconstruct *X* at the output layer, which is the same as the identity function *f*(*x*) = *x* (38). Then, the hidden layer is forced to learn a compressed representation of the data *X* from the input layer, which is reconstructed at the output layer as $\hat{X}$. The optimized model can be evaluated by the root mean squared error (RMSE) between *X* and $\hat{X}$.

In this study, we built a symmetric deep autoencoder model composed of *d*-dimensional input and output layers, and three hidden layers: *J* nodes for the second hidden layer (bottleneck), and 8 × *J* nodes for the first and third hidden layers. Also, a grid search using 1 ≤ *J* ≤ 10 was conducted to find the optimal number of nodes in the hidden layers. When compiling the model, RMSE and Adam were applied as the loss function and training optimizer, respectively. For the training process with 10-fold cross-validation, the batch size and the number of epochs were set to 64 and 100, respectively. Finally, representative nodes in the second hidden layer were used to extract features for the clustering process. We also conducted principal component analysis (PCA), one of the conventional feature extraction methods, before *k*-means clustering.

### *K*-means clustering

*K*-means clustering is an unsupervised machine learning algorithm (39). This algorithm is less computationally intensive for processing our large study data than hierarchical clustering. Also, the number of clusters (*k*) can be predefined by this algorithm to reveal our prior medical knowledge since the number of PI types is generally ≤ 10 in TEAM. Consider a *d*-dimensional data set *X* = {*x*_1_, *x*_2_, …, *x*_*n*_}, where *d* is the number of variables, this algorithm aims to partition the *n* observations into *k* (≤ *n*) sets *S* = {*S*_1_, *S*_2_, …, *S*_*k*_} to minimize the within-cluster sum of squares (WCSS). Formally, the objective is to find:


$$\begin{array}{l} \arg\;\underset{s}{\min}\sum_{i=1}^{k}\sum_{x\in S_{i}}||x-\mu_{i}|{|}^{2}\\ \left(\mu_{i}=mean\;of\;points\;in\;S_{i}\right) \end{array}$$

In this study, *k*-means clustering was performed on the data set using raw data and PCA-extracted and deep autoencoder-extracted features. The performance of the clusters was compared between each input type. We set the candidate number of clusters from *k* = 1 to *k* = 10, and 300 iterations for each *k* using the expectation-maximization style algorithm.

### Cluster evaluation

Cluster evaluation was conducted in two parts, internal cluster evaluation and external cluster evaluation. The Calinski-Harabasz index and silhouette coefficient were initially assessed for internal cluster evaluation (40). The optimal number of clusters was determined by the elbow method after plotting the WCSS with *k* values. All the above processes were conducted using the training set only. After determining the whole PI model including the feature extraction and clustering methods, the test set was inferred by the trained PI model. The PSQI, Berlin questionnaire, GSRS, and NQ scores, which were not used in feature extraction, were compared by external cluster evaluation.

### Statistical analysis

Summaries of the continuous variables are presented as means and standard deviations, and the categorical variables are presented as frequencies and percentages. For continuous variables, one-way analysis of variance (ANOVA) was used for comparing means among three clusters, followed by the Tukey-Kramer test for *post-hoc* multiple comparisons between two clusters with unequal sample sizes. For categorical variables, the chi-squared test was also performed. Statistical significance was set at *p* < 0.05.

### Tools

Python 3.8.0 (Python Software Foundation, Wilmington, DE, USA) was used for data preprocessing, model development and validation, visualization, and statistical analysis. The Python libraries Pandas and Numpy were adopted for data preprocessing; Scikit-learn was used for data preprocessing, PCA, and *k*-means clustering; Keras with Tensorflow backend for building and evaluating the deep autoencoder model; Statsmodels for statistical analysis of comparisons between clusters, and Seaborn with Matplotlib for data visualization. Google Colab, a cloud service for machine learning research, was used in this study. It provides various libraries and frameworks for deep learning and a robust graphics processing unit.

## Results

### Data set construction

**Figure 1:**
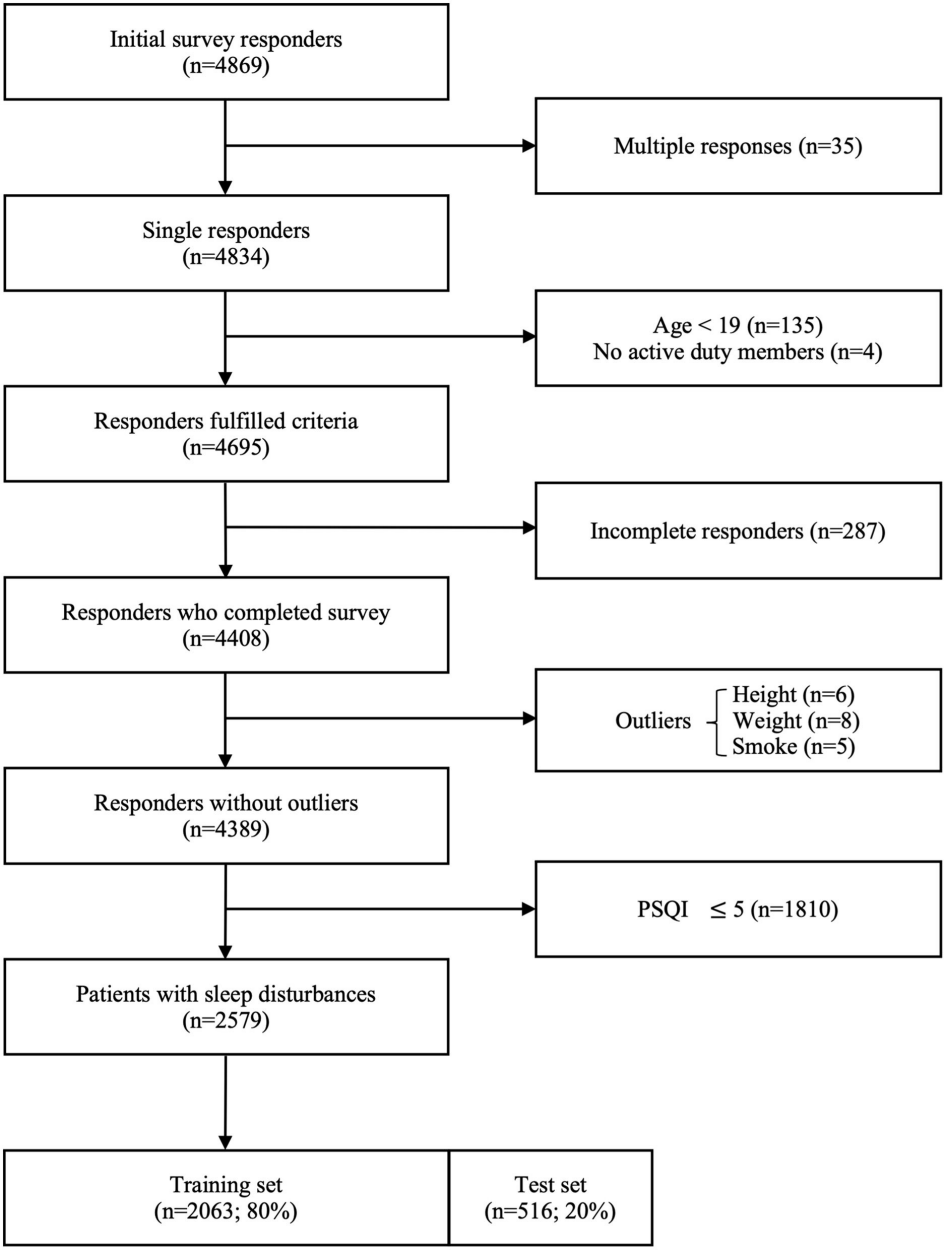
Flow chart illustrating the construction of the data set for the study.

Of a total of 4,869 survey responders, 35 multiple responders, and 139 responders who did not meet the inclusion criteria were excluded. A total of 4,408 responders completed the survey. After removing a few outliers for height (below 110 cm or above 200 cm), weight (below 40 kg or above 160 kg), smoking amount (above five packs per day), and smoking duration (above 20 years) through EDA, 4,389 responses remained. The data set of 2,579 patients with sleep disturbances was obtained after filtering by PSQI scores of >5, which were randomly split into a training set (*n* = 2,063; 80%) and a test set (*n* = 516; 20%). The flow chart of the data set construction process is shown in Figure 1.

### Feature extraction using PCA

**Figure 2:**
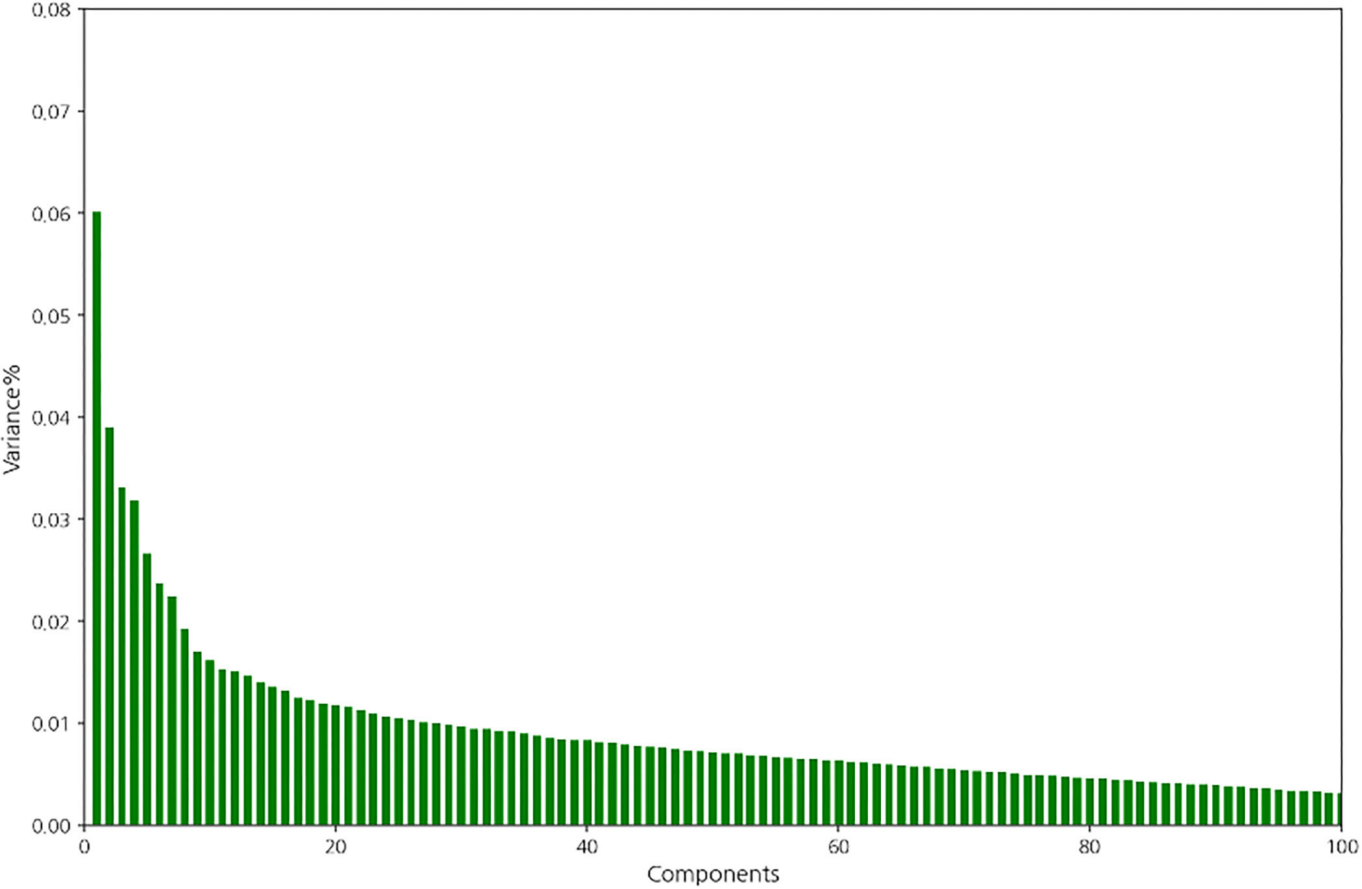
Variance of the components in the training set.

For comparison with the main feature extraction method, the deep autoencoder, PCA was first conducted using the training set. It showed that variance dropped off when the number of components was four, and the first four components explained the majority of the variance in the training set (Figure 2). Therefore, feature extraction using PCA was conducted with four components.

### Feature extraction using deep autoencoder

**Figure 3:**
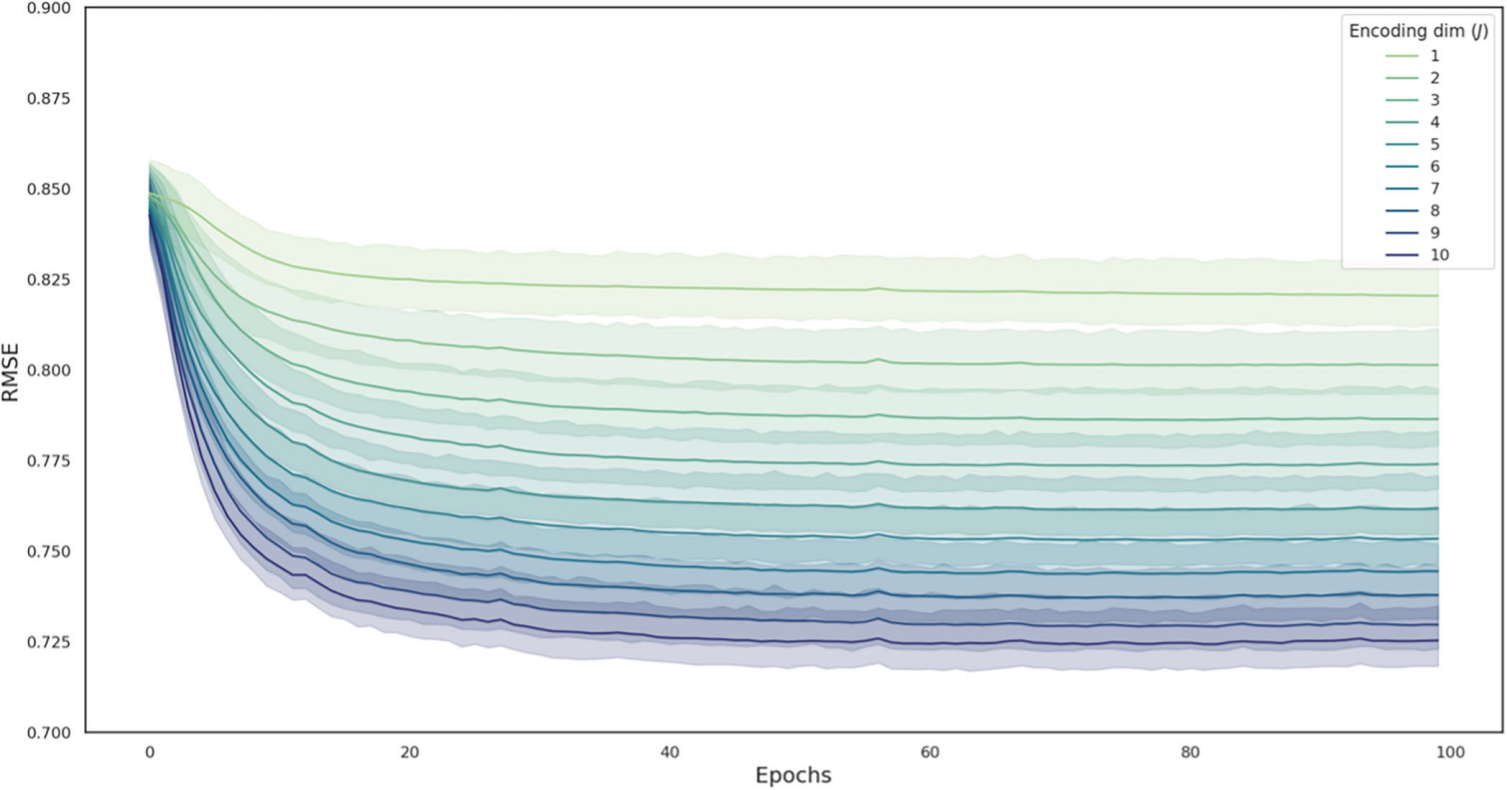
The change in RMSE during the model training process in each deep autoencoder architecture. Curves are averaged over 10 folds, with the shaded area representing the 95% confidence interval across folds. RMSE, root mean squared error.

Ten-fold cross-validation was conducted while training the deep autoencoder. The mean RMSE of the training set and validation set after 100 epochs (Table 1), and the change in RMSE of the validation set during training (Figure 3) are presented in each deep autoencoder architecture (the number of nodes in the second hidden layer).

**Table 1:** The mean RMSE for each model.

**The number of nodes in the second hidden layer (** ***J*** **)**	**RMSE**	
	**Training set**	**Validation set**
1	0.820 ± 0.004	0.821 ± 0.013
2	0.796 ± 0.002	0.802 ± 0.014
3	0.776 ± 0.002	0.787 ± 0.014
4	0.760 ± 0.002	0.774 ± 0.012
5	0.745 ± 0.003	0.763 ± 0.013
6	0.732 ± 0.003	0.752 ± 0.013
7	0.719 ± 0.003	0.744 ± 0.012
8	0.708 ± 0.004	0.737 ± 0.012
9	0.696 ± 0.004	0.732 ± 0.011
10	0.686 ± 0.004	0.725 ± 0.012

Values are presented as the mean ± standard deviation.

RMSE, root mean squared error.

### Internal cluster validation

**Figure 4:**
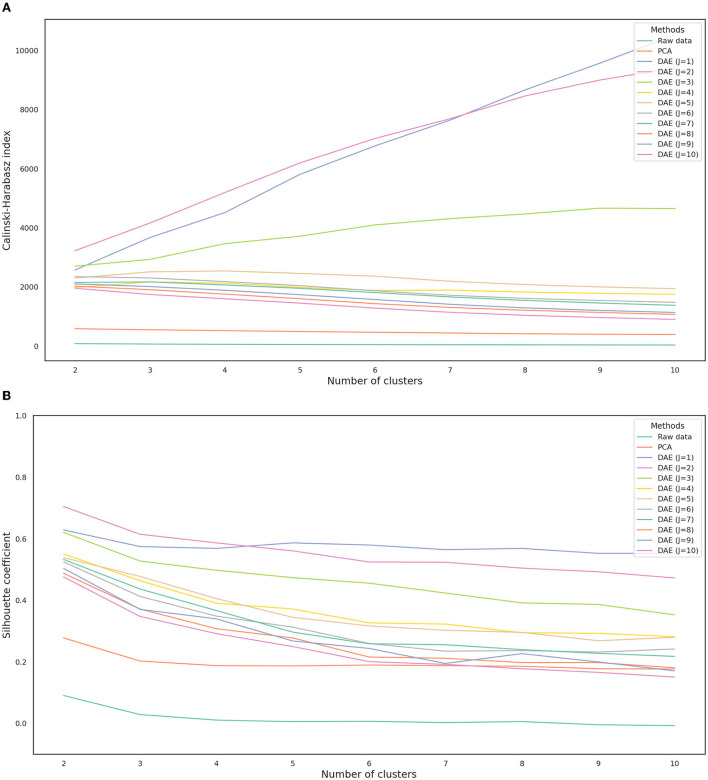
Calinski-Harabasz index **(A)** and silhouette coefficient **(B)** depending on each feature extraction method. *J* is the number of nodes in the second hidden layer. DAE, deep autoencoder; PCA, principal component analysis.

**Figure 5:**
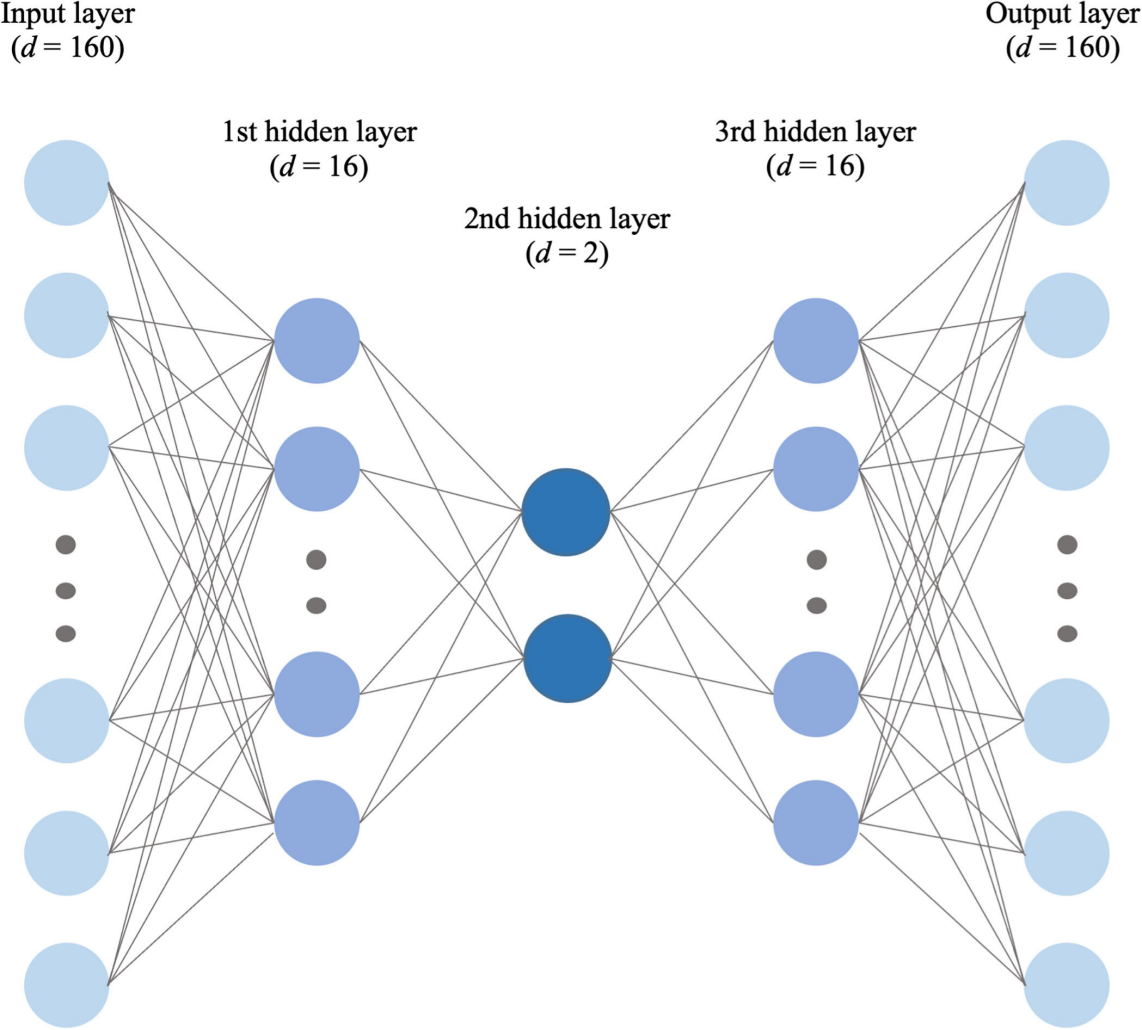
The final selected deep autoencoder model architecture for feature extraction.

**Figure 6:**
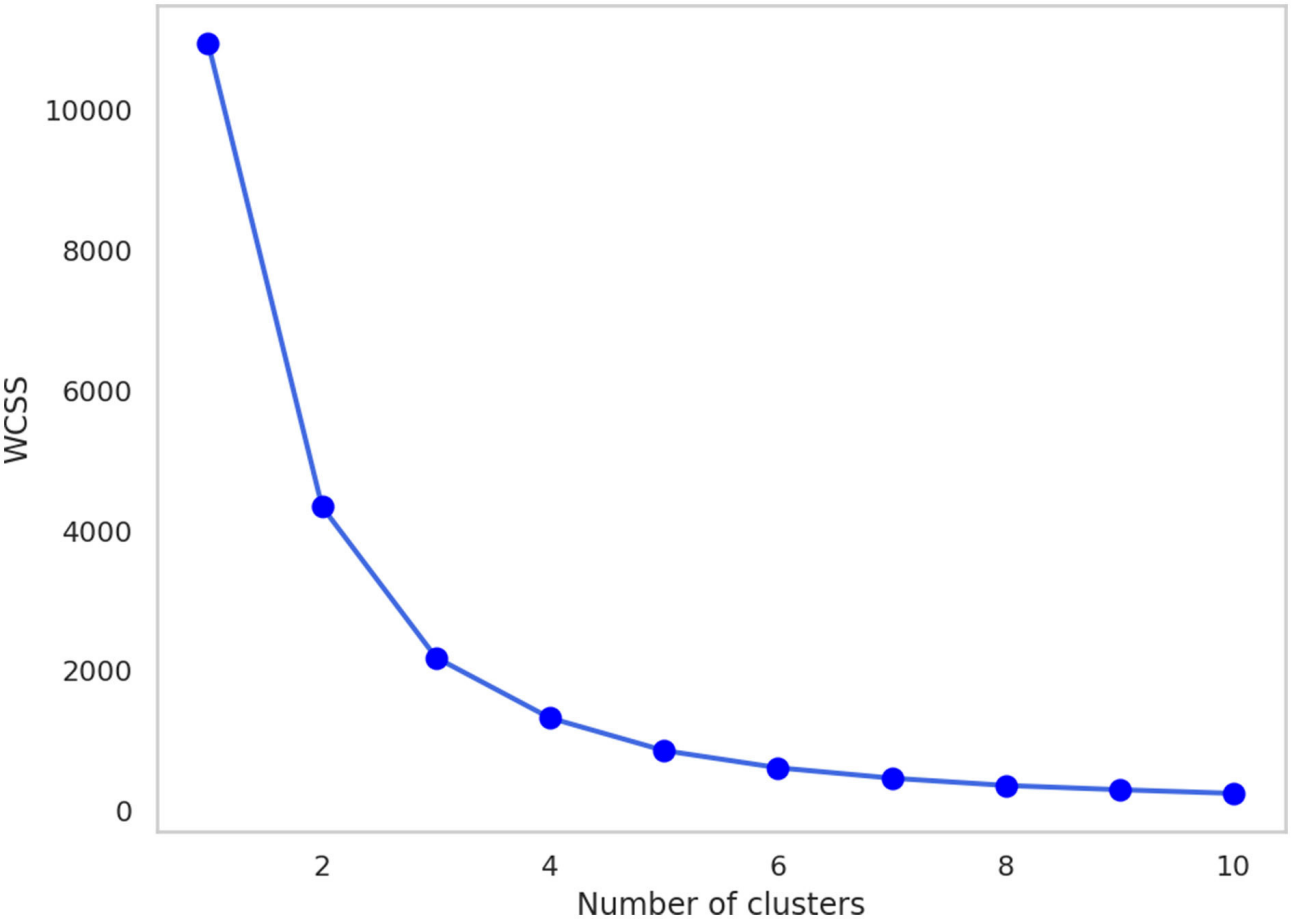
The change in WCSS with the number of clusters after feature extraction by the deep autoencoder (*J* = 2). WCSS, within-cluster sum of squares.

The Calinski-Harabasz index and silhouette coefficient after *k*-means clustering (2 ≤ *k* ≤ 10) are presented in Figure 4; Supplementary Table 1. The performance of clustering after feature extraction with the deep autoencoder was much better than that with raw data or PCA. Comparing the results of clustering after all feature extraction methods including PCA and the deep autoencoder in this study, the deep autoencoder (*J* = 2)—which presented the highest values of both the Calinski-Harabasz index and the silhouette coefficient in the small numbers of clusters (*k* ≤ 4)–might be the best feature extraction method for *k*-means clustering. The final deep autoencoder model architecture is shown in Figure 5. Also, considering both the Calinski-Harabasz index and the silhouette coefficient, *k* = 2 or 3 might be candidate clustering numbers. Finally, the optimal number (*k* = 3) of clusters was determined by the elbow method, a heuristic approach for determining the appropriate point for the local optimum (41, 42), as shown in Figure 6.

### External cluster validation

The patient characteristics in each cluster of the training set and test set are presented in Tables 2, 3 respectively. Among the clusters, the PSQI (*p* < 0.001), GSRS (*p* < 0.001), NQ balance (*p* = 0.008), NQ moderation (*p* < 0.001), NQ behavior (*p* < 0.001), and Berlin scores (*p* < 0.001) were significantly different in the training set, and PSQI (*p* < 0.001), GSRS (*p* < 0.001), NQ global (*p* < 0.001), NQ moderation (*p* < 0.001), and Berlin scores (*p* < 0.001) were significantly different in the test set (Table 4).

**Table 2:** Patient characteristics in each training set cluster.

**Characteristics**	**Cluster A** **(*****n*** = **1,396)**	**Cluster B** **(*****n*** = **98)**	**Cluster C** **(*****n*** = **569)**
Age, years (mean ± SD)	20.8 ± 1.2	21.2 ± 1.6	21.1 ± 1.4
Height, cm (mean ± SD)	174.6 ± 5.6	175.2 ± 6.0	174.8 ± 5.6
Weight, kg (mean ± SD)	72.2 ± 9.2	75.1 ± 11.1	73.8 ± 11.1
BMI, kg/m^2^ (mean ± SD)	23.6 ± 2.6	24.4 ± 3.2	24.1 ± 3.2
Smoking status			
Never, *n* (%)	739 (52.9)	53 (54.1)	244 (42.9)
Past, *n* (%)	84 (6.0)	4 (4.1)	44 (7.7)
Active, *n* (%)	573 (41.0)	41 (41.8)	281 (49.4)
Pack-years (mean± SD)	0.97 ± 1.95	1.65 ± 3.07	1.57 ± 2.48
Alcohol, *n* (%)			
<1 time/month	322 (23.1)	28 (28.6)	113 (19.9)
<1 time/week	396 (28.4)	26 (26.5)	130 (22.8)
1–2 times/week	493 (35.3)	25 (25.5)	199 (35.0)
3–7 times/week	185 (13.3)	19 (19.4)	127 (22.3)
Caffeine, *n* (%)			
Coffee			
<1 cup/week	511 (36.6)	34 (34.7)	193 (33.9)
1–2 cups/week	299 (21.4)	16 (16.3)	114 (20.0)
3–6 cups/week	252 (18.1)	15 (15.3)	90 (15.8)
1 cup/day	184 (13.2)	13 (13.3)	77 (13.5)
2 cups/day	102 (7.3)	11 (11.2)	59 (10.4)
≥3 cups/day	48 (3.4)	9 (9.2)	36 (6.3)
Energy drink			
<1 cup/week	907 (65.0)	46 (46.9)	335 (58.9)
1–2 cups/week	266 (19.1)	17 (17.3)	88 (15.5)
3–6 cups/week	113 (8.1)	19 (19.4)	69 (12.1)
1 cup/day	72 (5.2)	8 (8.2)	43 (7.6)
2 cups/day	22 (1.6)	3 (3.1)	23 (4.0)
≥3 cups/day	16 (1.1)	5 (5.1)	11 (1.9)
Bacchus ^#x000AE;^			
<1 cup/week	1,245 (90.9)	77 (78.6)	484 (85.1)
1–2 cups/week	121 (8.8)	13 (13.3)	60 (10.5)
3–6 cups/week	16 (1.2)	3 (3.1)	12 (2.1)
1 cup//day	10 (0.7)	2 (2.0)	7 (1.2)
2 cups/day	3 (0.2)	2 (2.0)	3 (0.5)
≥ 3 cups/day	1 (0.1)	1 (1.0)	3 (0.5)
Rank, *n* (%)			
Private	80 (5.7)	2 (2.0)	15 (1.2)
Private first class	606 (43.4)	38 (38.8)	211 (8.4)
Corporal	560 (40.1)	45 (45.9)	251 (11.6)
Sergeant	150 (12.9)	13 (13.3)	92 (3.7)
Education, *n* (%)			
Elementary school	1 (0.1)	0 (0.0)	0 (0.0)
Middle school	1 (0.1)	0 (0.0)	3 (0.5)
High school	1,326 (95.0)	89 (90.8)	518 (91.0)
University or college	68 (4.9)	9 (9.2)	48 (8.4)
Exercise, *n* (%)			
<1 day/week	217 (15.5)	27 (27.6)	121 (21.3)
1–2 days/week	331 (23.7)	20 (20.4)	140 (24.6)
3–4 days/week	399 (28.6)	27 (27.6)	162 (28.5)
≥5 days/week	449 (32.2)	24 (24.5)	146 (25.7)
Physical grade i*n* the military, *n* (%)			
First	413(29.6)	17 (17.3)	142 (25.0)
Second	596 (42.7)	43 (43.9)	224 (39.4)
Third	379 (27.1)	38 (38.8)	198 (34.8)
Fourth	4 (0.3)	0 (0.0)	3 (0.5)
Fifth or above	4 (0.3)	0 (0.0)	2 (0.4)
Sleep disorders, *n* (%)			
Present history			
None	1,306 (93.6)	65 (66.3)	470 (82.6)
Insomnia	37 (2.7)	16 (16.3)	50 (8.8)
Narcolepsy	30 (2.1)	17 (17.3)	27 (4.7)
Obstructive sleep apnea	5 (0.4)	6 (6.1)	12 (2.1)
Restless leg syndrome	7 (0.5)	2 (2.0)	14 (2.5)
Periodic limb movement	7 (0.5)	4 (4.1)	8 (1.4)
Past history			
None	1,353 (96.9)	80 (81.6)	526 (92.4)
Insomnia	32 (2.3)	11 (11.2)	31 (5.4)
Narcolepsy	3 (0.2)	8 (8.2)	3 (0.5)
Obstructive sleep apnea	4 (0.3)	2 (2.0)	7 (1.2)
Restless leg syndrome	2 (0.1)	1 (1.0)	4 (0.7)
Periodic limb movement	1 (0.1)	1 (1.0)	1 (0.2)
Gastrointestinal disorders, *n* (%)			
Present history			
None	1,337 (95.8)	67 (68.4)	487 (85.6)
Gastroesophageal reflux	23 (1.6)	19 (19.4)	41 (7.2)
Gastric ulcer	0 (0.0)	0 (0.0)	3 (0.5)
Duodenal ulcer	0 (0.0)	0 (0.0)	0 (0.0)
Irritable bowel syndrome	37 (2.7)	17 (17.3)	40 (7.0)
Past history			
None	1,263 (90.5)	63 (64.3)	440 (77.3)
Gastroesophageal reflux	74 (5.3)	26 (26.5)	74 (13.0)
Gastric ulcer	1 (0.1)	1 (1.0)	7 (1.2)
Duodenal ulcer	1 (0.1)	1 (1.0)	1 (0.2)
Irritable bowel syndrome	62 (4.4)	17 (17.3)	60 (10.5)
General diseases, *n* (%)			
None	1,322 (94.7)	85 (86.7)	527 (92.6)
Hypertension	65 (4.7)	12 (12.2)	34 (6.0)
Diabetes	5 (0.4)	2 (2.0)	2 (0.4)
Hyperlipidemia	4 (0.3)	1 (1.0)	5 (0.9)
Cardiac diseases	9 (0.6)	2 (2.0)	4 (0.7)
Medications, *n* (%)			
Sleeping pills	12 (0.9)	9 (9.2)	15 (2.6)
Sleep health supplements	5 (0.4)	5 (5.1)	12 (2.1)
Oral steroids	6 (0.4)	4 (4.1)	6 (1.1)
Melatonin	2 (0.1)	0 (0.0)	2 (0.4)
Anticonvulsants	0 (0.0)	0 (0.0)	2 (0.4)
Antidepressants	19 (1.4)	9 (9.2)	14 (2.5)
Beta blockers	1 (0.1)	0 (0.0)	0 (0.0)
Bronchodilators	4 (0.3)	2 (2.0)	5 (0.9)
Stimulants	3 (0.2)	4 (4.1)	6 (1.1)
Antihistamines	31 (2.2)	3 (3.1)	12 (2.1)
Weight loss pills	4 (0.3)	3 (3.1)	6 (1.1)
Weight loss supplements	26 (1.9)	8 (8.2)	21 (3.7)
Digestive pills	31 (2.2)	13 (13.3)	33 (5.8)
Digestive supplements	54 (3.9)	12 (12.2)	52 (9.1)
Stress^*^ (mean ± SD)	3.46 ± 1.57	3.41 ± 1.16	3.40 ± 1.44
Night shift with tomorrow duty-off			
Frequency, *n* (%)			
None	781 (55.9)	47 (48.0)	289 (50.8)
1 time/month	58 (4.2)	7 (7.1)	29 (5.1)
2 times/month	115 (8.2)	9 (9.2)	36 (6.3)
3 times/month	134 (9.6)	10 (10.2)	61 (10.7)
4 times/month	94 (6.7)	9 (9.2)	44 (7.7)
≥5 times/month	214 (15.3)	16 (16.3)	110 (19.3)
Sleep disturbance or fatigue^*^ (mean ± SD)	4.07 ± 0.87	4.43 ± 0.85	4.25 ± 0.89
Night shift without tomorrow duty-off			
Frequency, *n* (%)			
None	710 (50.9)	44 (44.9)	210 (36.9)
1 time/month	45 (3.2)	5 (5.1)	18 (3.2)
2 times/month	58 (4.2)	9 (9.2)	21 (3.7)
3 times/month	52 (3.7)	5 (5.1)	22 (3.9)
4 times/month	62 (4.4)	3 (3.1)	29 (5.1)
≥5 times/month	469 (33.6)	32 (32.7)	169 (29.7)
Sleep disturbance or fatigue^*^ (mean ± SD)	4.28 ± 0.85	4.50 ± 0.77	4.42 ± 0.85

Values are presented as the mean ± standard deviation (range) or number (%).

* Five-point Likert scale.

BMI, body mass index.

**Table 3:** Patient characteristics in each test set cluster.

**Characteristics**	**Cluster A** **(*****n*** = **352)**	**Cluster B** **(*****n*** = **22)**	**Cluster C** **(*****n*** = **142)**
Age, years (mean ± SD)	21.0 ± 1.4	21.9 ± 2.2	21.1 ± 1.5
Height, cm (mean ± SD)	174.2 ± 5.5	175.3 ± 4.7	174.9 ± 5.1
Weight, kg (mean ± SD)	72.5 ± 9.7	74.9 ± 7.8	74.6 ± 10.9
BMI, kg/m^2^ (mean ± SD)	23.8 ± 2.6	24.4 ± 2.5	24.4 ± 3.1
Smoking status			
Never, *n* (%)	186 (52.8)	6 (27.3)	60 (42.3)
Past, *n* (%)	19 (5.4)	3 (13.6)	11 (7.7)
Active, *n* (%)	147 (41.8)	13 (59.1)	71 (50.)
Pack-years (mean± SD)	0.94 ± 1.80	2.07 ± 2.99	1.78 ± 2.68
Alcohol, *n* (%)			
<1 time/month	77 (21.9)	6 (27.3)	25 (17.6)
<1 time/week	107 (30.4)	4 (18.2)	35 (24.6)
1–2 times/week	122 (34.7)	8 (36.4)	41 (28.9)
3–7 times/week	46 (13.1)	4 (18.2)	41 (28.9)
Caffeine, *n* (%)			
Coffee			
<1 cup/week	131 (37.2)	8 (36.4)	35 (24.6)
1–2 cups/week	89 (25.3)	2 (9.1)	31 (21.8)
3–6 cups/week	56 (15.9)	4 (18.2)	24 (16.9)
1 cup/day	44 (12.5)	6 (27.3)	24 (16.9)
2 cups/day	20 (5.7)	1 (4.5)	21 (14.8)
≥3 cups/day	12 (3.4)	1 (4.5)	7 (4.9)
Energy drink			
<1 cup/week	234 (66.5)	15 (68.2)	75 (52.8)
1–2 cups/week	65 (18.5)	3 (13.6)	37 (26.1)
3–6 cups/week	29 (8.2)	3 (13.6)	10 (7.0)
1 cup/day	13 (3.7)	1 (4.5)	11 (7.7)
2 cups/day	7 (2.0)	0 (0.0)	4 (2.8)
≥3 cups/day	4 (1.1)	0 (0.0)	5 (3.5)
Bacchus ^®^			
<1 cup/week	321 (91.2)	18 (81.8)	118 (83.1)
1–2 cups/week	27 (7.7)	3 (13.6)	12 (8.5)
3–6 cups/week	3 (0.9)	1 (4.5)	7 (4.9)
1 cup/day	0 (0.0)	0 (0.0)	3 (2.1)
2 cups/day	1 (0.3)	0 (0.0)	1 (0.7)
≥3 cups/day	0 (0.0)	0 (0.0)	1 (0.7)
Rank, *n* (%)			
Private	25 (7.1)	2 (9.1)	7 (4.9)
Private first class	142 (40.3)	3 (13.6)	48 (33.8)
Corporal	136 (38.6)	10 (45.5)	66 (46.5)
Sergeant	49 (13.9)	7 (31.8)	21 (14.8)
Education, *n* (%)			
Elementary school	0 (0.0)	0 (0.0)	0 (0.0)
Middle school	0 (0.0)	1 (4.5)	1 (0.7)
High school	329 (93.5)	19 (86.4)	134 (94.4)
University or college	23 (6.5)	2 (9.1)	7 (4.9)
Exercise, *n* (%)			
<1 day/week	50 (14.2)	6 (27.3)	23 (16.2)
1–2 days/week	91 (25.9)	5 (22.7)	35 (24.6)
3–4 days/week	88 (25.0)	5 (22.7)	36 (25.4)
≥5 days/week	123 (34.9)	6 (27.3)	48 (33.8)
Physical grade in the military, *n* (%)			
First	103 (29.3)	8 (36.4)	31 (21.8)
Second	141 (40.1)	6 (27.3)	63 (44.4)
Third	107 (30.4)	7 (31.8)	47 (33.1)
Fourth	1 (0.3)	1 (4.5)	1 (0.7)
Fifth or above	0 (0.0)	0 (0.0)	0 (0.0)
Sleep disorders, *n* (%)			
Present history			
None	334 (90.9)	18 (81.8)	114 (80.3)
Insomnia	10 (2.8)	3 (13.6)	14 (9.9)
Narcolepsy	5 (1.4)	2 (9.1)	7 (4.9)
Obstructive sleep apnea	1 (0.3)	0 (0.0)	2 (1.4)
Restless leg syndrome	1 (0.3)	1 (4.5)	6 (4.2)
Periodic limb movement	2 (0.6)	1 (4.5)	5 (3.5)
Past history			
None	344 (97.7)	20 (90.9)	131 (92.3)
Insomnia	5 (1.4)	2 (9.1)	6 (4.2)
Narcolepsy	2 (0.6)	0 (0.0)	3 (2.1)
Obstructive sleep apnea	0 (0.0)	0 (0.0)	1 (0.7)
Restless leg syndrome	1 (0.3)	0 (0.0)	1 (0.7)
Periodic limb movement	0 (0.3)	0 (0.0)	1 (0.7)
Gastrointestinal disorders, *n* (%)			
Present history			
None	329 (93.5)	20 (90.9)	130 (91.5)
Gastroesophageal reflux	13 (3.7)	2 (9.1)	3 (2.1)
Gastric ulcer	0 (0.0)	0 (0.0)	0 (0.0)
Duodenal ulcer	0 (0.0)	0 (0.0)	0 (0.0)
Irritable bowel syndrome	12 (3.4)	0 (0.0)	10 (7.0)
Past history			
None	300 (85.2)	15 (68.2)	119 (83.8)
Gastroesophageal reflux	22 (6.3)	5 (22.7)	11 (7.7)
Gastric ulcer	5 (1.4)	1 (4.5)	0 (0.0)
Duodenal ulcer	1 (0.3)	0 (0.0)	0 (0.0)
Irritable bowel syndrome	27 (7.7)	3 (13.6)	14 (9.9)
General diseases, *n* (%)			
None	331 (94.0)	12 (54.5)	129 (90.8)
Hypertension	18 (5.1)	5 (22.7)	10 (7.0)
Diabetes	1 (0.3)	0 (0.0)	2 (1.4)
Hyperlipidemia	3 (0.9)	0 (0.0)	2 (1.4)
Cardiac diseases	1 (0.3)	2 (9.1)	2 (1.4)
Medications, *n* (%)			
Sleeping pills	0 (0.0)	1 (4.5)	3 (2.1)
Sleep health supplements	1 (0.3)	0 (0.0)	1 (0.7)
Oral steroids	2 (0.6)	1 (4.5)	1 (0.7)
Melatonin	0 (0.0)	1 (4.5)	0 (0.0)
Anticonvulsants	0 (0.0)	0 (0.0)	0 (0.0)
Antidepressants	2 (0.6)	2 (9.1)	3 (2.1)
Beta blockers	0 (0.0)	0 (0.0)	0 (0.0)
Bronchodilators	1 (0.3)	0 (0.0)	1 (0.7)
Stimulants	1 (0.3)	0 (0.0)	0 (0.0)
Antihistamines	10 (2.8)	1 (4.5)	4 (2.8)
Weight loss pills	0 (0.0)	0 (0.0)	0 (0.0)
Weight loss supplements	3 (0.9)	1 (4.5)	10 (7.0)
Digestive pills	9 (2.6)	2 (9.1)	6 (4.2)
Digestive supplements	12 (3.4)	2 (9.1)	9 (6.3)
Stress^*^ (mean ± SD)	3.57 ± 1.57	3.41 ± 1.30	3.42 ± 1.38
Night shift with tomorrow duty-off			
Frequency, *n* (%)			
None	192 (54.5)	10 (45.5)	72 (50.7)
1 time/month	19 (5.4)	3 (13.6)	7 (4.9)
2 times/month	36 (10.2)	2 (9.1)	7 (4.9)
3 times/month	32 (9.1)	3 (13.6)	13 (9.2)
4 times/month	21 (6.0)	2 (9.1)	7 (4.9)
≥5 times/month	52 (14.8)	2 (9.1)	36 (25.4)
Sleep disturbance or fatigue^*^ (mean ± SD)	3.96 ± 0.89	4.13 ± 0.74	4.43 ± 0.80
Night shift without tomorrow duty-off			
Frequency, *n* (%)			
None	175 (49.7)	10 (45.5)	84 (59.2)
1 time/month	19 (5.4)	2 (9.1)	6 (4.2)
2 times/month	13 (3.7)	2 (9.1)	5 (3.5)
3 times/month	15 (4.3)	1 (4.5)	4 (2.8)
4 times/month	16 (4.5)	0 (0.0)	13 (9.2)
≥5 times/month	114 (32.4)	7 (31.8)	30 (21.1)
Sleep disturbance or fatigue^*^ (mean ± SD)	4.24 ± 0.88	4.67 ± 0.62	4.42 ± 0.76

Values are presented as the mean ± standard deviation (range) or number (%).

* Five-point Likert scale.

BMI, body mass index.

**Table 4:** Results of external cluster validation in the training set and test set.

	**Cluster A**	**Cluster B**	**Cluster C**			**Cluster A vs. B**		**Cluster A vs. C**		**Cluster B vs. C**	
	**mean ± SD or ratio**	**mean ± SD or ratio**	**mean ± SD or ratio**	**F/** *X* ^2^	***p-*** **value**	**Difference or OR [95% CI]**	***p-*** **value**	**Difference or OR [95% CI]**	***p-*** **value**	**Difference or OR [95% CI]**	***p-*** **value**
**Training set**	*n* = 1,396	*n* = 98	*n* = 569						
PSQI	8.33 ± 2.20	11.57 ± 3.31	9.96 ± 2.81	149.27	<0.001	−3.24 [−3.84, −2.64]	<0.001	−1.62 [−1.91, −1.34]	<0.001	1.62 [0.99, 2.24]	<0.001
GSRS	2.18 ± 2.15	17.94 ± 7.38	6.39 ± 4.09	1,305.82	<0.001	−15.76 [−16.55, −14.98]	<0.001	−4.21 [−4.59, −3.84]	<0.001	11.55 [10.73, 12.37]	<0.001
NQ global	41.51 ± 8.78	42.78 ± 11.24	42.11 ± 10.65	1.45	0.234	−1.27 [−3.59, 1.05]	0.404	−0.60 [−1.71, 0.50]	0.406	0.67 [−1.76, 3.09]	0.774
NQ balance	35.44 ± 14.33	33.25 ± 15.37	33.24 ± 16.73	4.79	0.008	2.18 [−1.51, 5.88]	0.349	2.20 [0.44, 3.96]	0.010	0.02 [−3.85, 3.88]	0.900
NQ diversity	12.67 ± 4.21	11.95 ± 4.54	12.83 ± 4.55	1.76	0.172	0.73 [−0.33, 1.79]	0.240	−0.16 [−0.66, 3.48]	0.728	−0.89 [−1.99, 0.22]	0.147
NQ moderation	8.65 ± 4.18	12.9 ± 5.85	11.12 ± 4.91	90.63	<0.001	−4.25 [−5.35, −3.15]	<0.001	−2.48 [−3.00, −1.95]	<0.001	1.77 [0.62, 2.92]	<0.001
NQ behavior	11.38 ± 3.46	9.67 ± 3.74	9.90 ± 3.66	42.07	<0.001	1.72 [0.85, 2.58]	<0.001	1.49 [1.08, 1.90]	<0.001	−0.23 [−1.13, 0.68]	0.806
Berlin score (low/high)	1,230/166	63/35	391/178	122.00	<0.001	0.24 [0.16, 0.38]	<0.001	0.30 [0.23, 0.38]	<0.001	1.22 [0.78, 1.91]	0.452
**Test set**	n=352	n=22	n=142								
PSQI	8.36 ± 2.12	11.14 ± 3.45	10.04 ± 2.84	34.32	<0.001	−2.78 [−4.02, −1.53]	<0.001	−1.68 [−2.24, −1.12]	<0.001	1.09 [−0.20, 2.39]	0.117
GSRS	2.08 ± 2.05	19.77 ± 8.15	6.52 ± 3.73	406.57	<0.001	−17.69 [−19.28, −16.10]	<0.001	−4.44 [−5.16, −3.72]	<0.001	13.25 [11.60, 14.91]	<0.001
NQ global	40.30 ± 8.28	44.71 ± 17.48	43.83 ± 10.59	8.31	<0.001	−4.41 [−9.31, 0.50]	0.089	−3.52 [−5.74, −1.30]	<0.001	0.88 [−4.23, 6.00]	0.900
NQ balance	33.89 ± 13.10	37.55 ± 24.27	35.30 ± 17.34	0.95	0.387	−3.66 [−11.39, 4.08]	0.508	−1.42 [−4.92, 2.08]	0.599	2.24 [−5.82, 10.30]	0.770
NQ diversity	12.33 ± 4.05	11.62 ± 5.69	12.88 ± 4.12	1.34	0.263	0.72 [−1.43, 2.86]	0.694	−0.54 [−1.51, 0.43]	0.386	−1.26 [−3.49, 0.97]	0.382
NQ moderation	8.43 ± 4.02	12.32 ± 5.49	11.71 ± 4.88	37.53	<0.001	−4.89 [−7.13, −2.64]	<0.001	−3.28 [−4.29, −2.26]	<0.001	1.61 [−0.73, 3.95]	0.238
NQ behavior	11.12 ± 3.41	10.42 ± 4.12	10.47 ± 3.91	1.90	0.151	0.70 [−1.15, 2.55]	0.639	0.65 [−0.18, 1.49]	0.159	−0.04 [−1.97, 1.89]	0.900
Berlin score (low/high)	305/47	14/8	100/42	22.09	<0.001	0.27 [0.11, 0.68]	0.008	0.37 [0.23, 0.59]	<0.001	1.36 [0.53, 3.48]	0.693

GSRS, gastrointestinal symptom rating scale; NQ, nutrition quotient; OR, odds ratio; PSQI, Pittsburgh sleep quality index; SD, standard deviation.

Through *post-hoc* analysis (Table 4), the mean PSQI score of cluster A was significantly lower than that of cluster B (−3.24, 95% confidence interval (CI) −3.84, −2.64], *p* < 0.001) and cluster C (−1.62, 95% CI [−1.91, −1.34], *p* < 0.001) in the training set. The mean PSQI score of cluster B was significantly higher than that of cluster C (1.62, 95% CI [0.99, 2.24], *p* < 0.001) in the training set. The mean PSQI score of cluster A was significantly lower than that of cluster B (−2.78, 95% CI [−4.02, −1.53], *p* < 0.001) and cluster C (−1.68, 95% CI [−2.24, −1.12], *p* < 0.001) in the test set. The mean PSQI score of cluster B was also higher but not significantly different than that of cluster C (1.09, 95% CI [−0.20, 2.39], *p* = 0.117) in the test set.

The mean GSRS score of cluster A was significantly lower than that of cluster B (−15.76, 95% CI [−16.55, −14.98], *p* < 0.001) and cluster C (−4.21, 95% CI [−4.59, −3.84], *p* < 0.001) in the training set. The mean GSRS score of cluster B was significantly higher than that of cluster C (11.55, 95% CI [10.73, 12.37], *p* < 0.001) in the training set. The mean GSRS score of cluster A was significantly lower than that of cluster B (−17.69, 95% CI [−19.28, −16.10], *p* < 0.001) and cluster C (−4.44, 95% CI [−5.16, −3.72], *p* < 0.001) in the test set. The mean GSRS score of cluster B was significantly higher than that of cluster C (13.25, 95% CI [11.60, 14.91], *p* < 0.001) in the test set.

The mean NQ global score of cluster A was lower but not statistically different from that of cluster B (−1.27, 95% CI [−3.59, 1.05], *p* = 0.404) and cluster C (−0.60, 95% CI [−1.71, 0.50], *p* = 0.406) in the training set. The mean NQ global score of cluster B was higher but not significantly different than that of cluster C (0.67, 95% CI [−1.76, 3.09], *p* = 0.774) in the training set. The mean NQ global score of cluster A was lower but not statistically different than that of cluster B (−4.41, 95% CI [−9.31, 0.50], *p* = 0.089) and significantly lower than that of cluster C (−3.52, 95% CI [−5.74, −1.30], *p* < 0.001) in the test set. The mean NQ global score of cluster B was higher but not significantly different than that of cluster C (0.88, 95% CI [−4.23, 6.00], *p* = 0.900) in the test set.

The mean NQ balance score of cluster A was higher but not statistically different than that of cluster B (2.18, 95% CI [−1.51, 5.88], *p* = 0.349) and significantly higher than that of cluster C (2.20, 95% CI [0.44, 3.96], *p* = 0.010) in the training set. The mean NQ balance score of cluster B was higher but not significantly different than that of cluster C (0.02, 95% CI [−3.85, 3.88], *p* = 0.900) in the training set. The mean NQ balance score of cluster A was lower but not statistically different than that of cluster B (−3.66, 95% CI [−11.39, 4.08], *p* = 0.508) and cluster C (−1.42, 95% CI [−4.92, 2.08], *p* = 0.599) in the test set. The mean NQ balance score of cluster B was higher but not significantly different than that of cluster C (2.24, 95% CI [−5.82, 10.30], *p* = 0.770) in the test set.

The mean NQ diversity score of cluster A was higher but not statistically different than that of cluster B (0.73, 95% CI [−0.33, 1.79], *p* = 0.240) and lower but not statistically different than that of cluster C (−0.16, 95% CI [−0.66, 3.48], *p* = 0.728) in the training set. The mean NQ diversity score of cluster B was lower but not significantly different than that of cluster C (−0.89, 95% CI [−1.99, 0.22], *p* = 0.147) in the training set. The mean NQ diversity score of cluster A was higher but not statistically different than that of cluster B (0.72, 95% CI [−1.43, 2.86], *p* = 0.694) and lower but not statistically different than that of cluster C (−0.54, 95% CI [−1.51, 0.43], *p* = 0.386) in the test set. The mean NQ diversity score of cluster B was lower but not significantly different than that of cluster C (−1.26, 95% CI [−3.49, 0.97], *p* = 0.382) in the test set.

The mean NQ moderation score of cluster A was significantly lower than that of cluster B (−4.25, 95% CI [−5.35, −3.15], *p* < 0.001) and cluster C (−2.48, 95% CI [−3.00, −1.95], *p* < 0.001) in the training set. The mean NQ moderation score of cluster B was significantly higher than that of cluster C (1.77, 95% CI [0.62, 2.92], *p* < 0.001) in the training set. The mean NQ moderation score of cluster A was significantly lower than that of cluster B (−4.89, 95% CI [−7.13, −2.64], *p* < 0.001) and cluster C (−3.28, 95% CI [−4.29, −2.26], *p* < 0.001) in the test set. The mean NQ moderation score of cluster B was also higher but not significantly different than that of cluster C (1.61, 95% CI [−0.73, 3.95], *p* = 0.238) in the test set.

The mean NQ behavior score of cluster A was significantly higher than that of cluster B (1.72, 95% CI [0.85, 2.58], *p* < 0.001) and cluster C (1.49, 95% CI [1.08, 1.90], *p* < 0.001) in the training set. The mean NQ behavior score of cluster B was lower but not significantly different than that of cluster C (−0.23, 95% CI [−1.13, 0.68], *p* = 0.806) in the training set. The mean NQ behavior score of cluster A was higher but not significantly different than that of cluster B (0.70, 95% CI [−1.15, 2.55], *p* = 0.639) and cluster C (0.65, 95% CI [−0.18, 1.49], *p* = 0.159) in the training set. The mean NQ behavior score of cluster B was lower but not significantly different than that of cluster C (−0.04, 95% CI [−1.97, 1.89], *p* = 0.900) in the test set.

The Berlin score showed that cluster A had a significantly lower risk of OSA than that of cluster B (odds ratio (OR) = 0.24, 95% CI [0.16, 0.38], *X*^2^ = 42.61, *p* < 0.001) and cluster C (OR = 0.30, 95% CI [0.23, 0.38], *X*^2^ = 103.92, *p* < 0.001) in the training set. The Berlin score showed that cluster B had a higher risk than that of cluster C (OR = 1.22, 95% CI [0.78, 1.91], *X*^2^ = 0.57, *p* = 0.452) in the training set. The Berlin score showed that cluster A had a significantly lower risk of OSA than that of cluster B (OR = 0.27, 95% CI [0.11, 0.68], *X*^2^ = 7.00, *p* = 0.008) and cluster C (OR = 0.37, 95% CI [0.23, 0.59], *X*^2^ = 16.95, *p* < 0.001) in the test set. The Berlin score showed that cluster B had a higher risk than that of cluster C (OR = 1.36, 95% CI [0.53, 3.48], *X*^2^ = 0.16, *p* = 0.693) in the test set.

**Figure 7:**
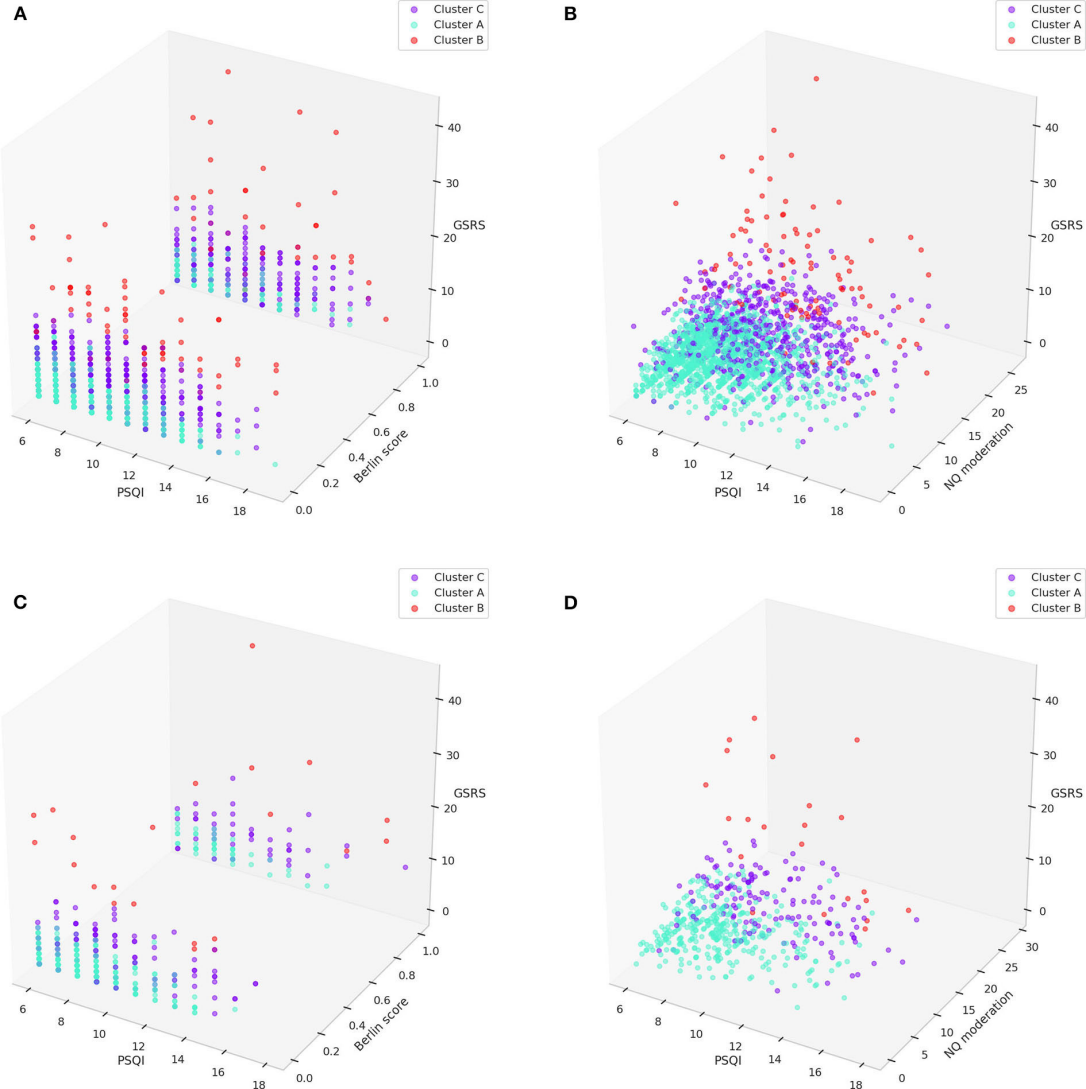
3D visualization of clusters with major components (PSQI, Berlin, GSRS scores, and NQ moderation) in the training set **(A,B)** and test set **(C,D)**. GSRS, gastrointestinal symptom rating scale; NQ, nutrition quotient; PSQI, Pittsburgh sleep quality index.

Three–dimensional clustering visualizations were presented with the major components that were statistically different by multi-comparison and *post-hoc* analysis in both the training and test sets, and statistically different by multi-comparison only in both the training and test sets; NQ moderation between cluster B and C was not statistically different by *post-hoc* analysis in the test set (Figure 7).

## Discussion

This study demonstrated that the deep autoencoder method was a better feature extraction method for the clustering of sleep disturbances than PCA. This result is comparable to that of other studies in that the autoencoder effectively reduces the high-dimensionality of the various types of data since it can learn non-linear feature representations (43–45). Specifically, based on internal cluster validation and the elbow method, the best architecture of the deep autoencoder for extracting features for clustering our study samples with sleep disturbances was 16 nodes for the first and third hidden layers, and two nodes for the second hidden layer, while the optimal number of clusters was considered to be three. After external cluster validation, three PI types were differentiated by changes in sleep quality, dietary habits, and concomitant gastrointestinal symptoms.

**Figure 8:**
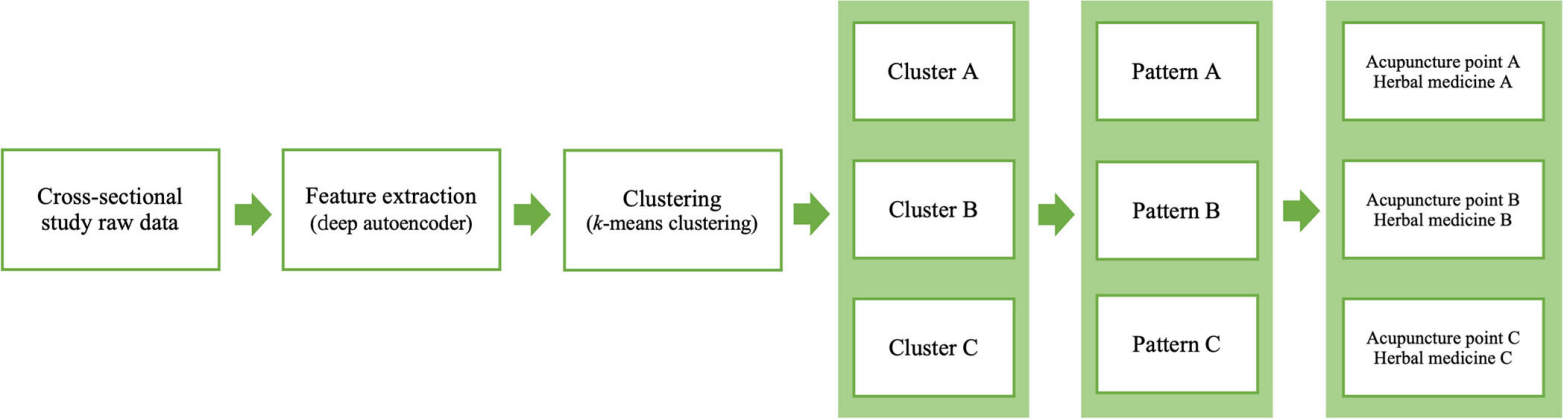
Flow diagram of the proposed PI model for acupuncture point and herbal medicine selection.

PI has been used in TEAM for the personalized care of various conditions including sleep disorders. As the accurate diagnosis and precise evaluation of individual patients are the key for personalized care in conventional medicine, PI, as well as diagnosis according to the International Classification of Diseases, Tenth Revision (ICD-10), is an important principle in personalized TEAM treatments such as acupuncture point selections and combinations of herbal medicines. Although several previous studies have tried to standardize PI and suggested new methods for PI in different types of data, it is considered a “black box” in which the external validity or usability in clinical TEAM practice cannot be ensured (14, 15, 46–48). Therefore, in another aspect of PI standardization, we proposed a new paradigm, the clinical data-driven PI model, applying advanced machine learning techniques. The PI model is flexible in the data characteristics that can be used and is reproducible for certain data to enhance the effectiveness of TEAM treatment in clinical practice (Figure 8).

There were three main aspects to this study, data type, feature extraction, and clustering. First, whole raw data from a cross-sectional study were used. The cross-sectional study data were generally composed of fundamental clinical information such as age, sex, and medical history, and symptoms, and/or a disease-related questionnaire. Particularly, our used cross-sectional study included several questionnaires with different domains including sleep, diet, nutrition, and gastrointestinal status. Since TEAM doctors usually ask not only about sleep conditions but also about other conditions to select the appropriate acupuncture points and/or herbal medicines for treating insomnia patients (28, 30), this type of data was suitable for reflecting clinical settings. Furthermore, this data type may assure external cluster validation. Clustering validation, which measures the goodness of clustering results, can be categorized into two methods: internal cluster validation and external cluster validation (49). The internal cluster validation is conducted without the need to obtain any additional information, such as evaluating the average between- and within-cluster sums of squares (Calinski-Harabasz index), or the difference of the between- and within-cluster distances (silhouette coefficient). On the other hand, the external cluster validation is conducted with other external information, such as a true class of cluster or previous knowledge about data. In this study, instead of obtaining the true labels of each cluster, which require large amounts of cost and time for TEAM doctors, we used the final scores of PSQI, NQ, GSRS, and the Berlin score, which were not used in the input features of clustering, but which could be calculated using specific non-linear functions respectively to externally compare the clustering results.

Second, feature extraction was conducted by a deep autoencoder model. Two methods have been used before the clustering process, feature selection (selecting a small subset of actual features from the data) and feature extraction (constructing a small set of artificial features from the data). Most clinical studies conducted feature selection through statistical methods such as the *t*-test or chi-squared test between two groups or it was determined by clinical experience or medical knowledge. However, in a large series of data, so-called high-dimensional data, it was difficult to find the best feature selection strategy for efficiently reducing the dimension of the data (50). Therefore, some algorithms such as PCA and the autoencoder have been suggested for feature extraction (51), very similar to a TEAM doctor's PI process made by observing patients with not just a few pieces of clinical information but comprehensively, using a lot of clinical information. This characteristic of TEAM doctors' decision-making might also be related to the reason why deep autoencoder model extraction was much more efficient than that of other methods in our study. As decision-making in TEAM is complex and the interactions between clinical information and PI are non-linear, autoencoder architecture learning non-linear mapping allows for the transformation of high-dimensional data into more clustering-friendly representations, whereas PCA is fundamentally limited to linear embedding, and it is possible to lose essential features (38). Another strength of using the deep autoencoder for feature extraction is that it can extract features from non-quantizable questionnaire responses (e.g., dietary habit survey questionnaire), which does not use a formula to generate a single score, and efficiently prevents the curse of dimensionality without suffering from high computational complexity in large-scale data (38).

Third, *k*-means clustering, an unsupervised machine learning algorithm serving as a powerful computational method to analyze high-dimensional data in the form of sequences or expressions, was used in this study (38). It does not need data labeling, which is costly and time-consuming in biomedical research using supervised learning. In addition, even if data labeling is performed by several TEAM doctors, the labeling results are highly likely to be inconsistent because the types of PI are inconsistent among TEAM doctors and each TEAM society and different depending upon the disease. Therefore, a data-driven approach to PI for TEAM research, which is flexible for changes in data and reproducible for certain data, might be more reasonable than a standardization approach using a few TEAM research experts.

Each cluster of sleep disturbance patients could be differentiated, as shown in Figure 7. The patients in cluster A had relatively mild sleep disturbances, severe immoderation in the amount of food consumed, and good gastrointestinal status compared to the other clusters. The patients in cluster B had relatively severe sleep disturbances, mild immoderation in the amount of food consumed, and severe gastrointestinal problems compared to the other clusters. The patients in cluster C had relatively moderate sleep disturbances, moderate immoderation in the amount of food consumed, and mild-to-moderate gastrointestinal problems compared to the other clusters. Although the statistical analysis of the Berlin score indicated that cluster A had a much lower risk than the other two clusters, it could not be observed in the 3-dimensional visualizations.

The clustering results can be interpreted in two aspects, the changes in sleep quality and the concomitant symptoms. As sleep quality deteriorates, the appetite associated with food moderation decreases, and the condition of the gastrointestinal system worsens. Based on a recent systematic review and meta-analysis of acupuncture using PI and TEAM clinical guidelines for insomnia patients, cluster A may be matched to the “stomach disharmony pattern” type using ST36, CV12, and ST25; cluster C may be matched to the “pattern of lingering phlegm” type using ST40 and CV12; and cluster B may be matched to the “pattern of dual deficiency of heart and spleen” type using CV12, ST36, and ST40 (29, 30). This clustering model can automatically and consistently provide the same PI for a certain patient, which ensures reliability for both TEAM doctors and patients. However, it should be noted that this clustering model is flexible to the number of patient data, changes in patient features, or changes in the target disease, so-called “transfer learning” and “fine-tuning” in machine learning techniques (52), which might provide a different output for the number or types of patterns identified. Therefore, the novel PI model in the present study can be advanced, modified, or expanded for other studies.

The applications of this study include AI-based clinical decision support systems (CDSSs) through electronic medical records (EMRs) and clinical trial protocols for evaluating the effectiveness of TEAM treatment. If a TEAM doctor in clinical practice obtains clinical data from insomnia patients and documents them in the EMR, the PI model in AI-based CDSSs suggests the candidate PI with the associated probability and recommends a fundamental combination of acupuncture points and herbal medicines. In addition, most pragmatic trial protocols with individualized TEAM treatment depend completely upon (one person or more) the TEAM doctor's PI for each patient. The reliability and validity of PI itself, which might affect the effect size of individualized TEAM treatments, are limited. However, the PI model in this study could suggest a consistent PI technique for patients with similar features, although the model's effect on the results of individualized TEAM treatment should be validated in a prospective clinical trial.

Some limitations of this study follow. First, this cross-sectional study data might not be fully sufficient to mimic the interaction between doctors and patients in clinical practices. Some data obtained from free medical notes or an AI speaker in clinical settings might be helpful to overcome this limitation. Second, since this data was obtained from a single sample of sleep disturbances in the ROKA, another study sample is required for external validation of our proposed model. Third, this study sequentially used a feature extraction model and a clustering model separately. Emerging machine learning research such as a deep clustering network, which optimizes the feature extraction model and the clustering model simultaneously, might perform better than the techniques used in our study. This will be considered in future studies. Fourth, the PI data used for each patient made by TEAM doctors were limited in this study. However, the correlation between our model's output and actual PI by TEAM doctors in this study should be observed to externally and more robustly validate our clustering results. Fifth, although all features of data were included to reflect a clinical setting wherein TEAM doctors might consider all information of patients as much as possible to find the appropriate PI, the feature selection algorithms, such as univariate statistical test, Lasso regularization, or Boruta algorithm can be applied in future studies to improve upon our results. Finally, the specific combinations of acupuncture points and herbal medicines after PI process were not represented in this study. Although this study revealed the basic concepts of the novel data-driven PI model, more research such as a systematic review of published clinical articles, including case series, or a survey of TEAM doctors is required to recommend the appropriate acupuncture points and/or herbal medicines after the determination of PI.

## Data availability statement

The raw data supporting the conclusions of this article will be made available by the authors, without undue reservation.

## Code availability

Code for this paper is provided at https://github.com/HyeonhoonLee/DeepPI.

## Ethics statement

The studies involving human participants were reviewed and approved by Institutional Review Board of the Armed Forces Medical Command (AFMC-202107-HR-054-02). The patients/participants provided their written informed consent to participate in this study.

## Author contributions

HL and YC designed the research study. CY and J-DL supervised the study. HL, YC, BS, and SL collected and analyzed the survey data. HL drafted the manuscript. SL, JK, KK, and EK provided critical comments for improvement of the manuscript. All authors contributed to the article and approved the submitted version.

## Funding

This research was supported by grants from Korea Institute of Oriental Medicine (KSN2022210).

## Conflict of interest

The authors declare that the research was conducted in the absence of any commercial or financial relationships that could be construed as a potential conflict of interest.

## Publisher's note

All claims expressed in this article are solely those of the authors and do not necessarily represent those of their affiliated organizations, or those of the publisher, the editors and the reviewers. Any product that may be evaluated in this article, or claim that may be made by its manufacturer, is not guaranteed or endorsed by the publisher.
