# Supplementary material for: Deep autoencoder-powered pattern identification of sleep disturbance using multi-site cross-sectional survey data
Source: Front Med (Lausanne). 2022 Jul 29;9:950327. doi: 10.3389/fmed.2022.950327 (PMC9374171; doi:10.3389/fmed.2022.950327)
Supplement: Supplementary file 1 [file Table_1.docx]

| **Supplement 1: The results of internal cluster validation**  **Supplementary Table 1. Calinski-Harabasz index** | | | | | |  | |  | |  |  | |  | |  |
| --- | --- | --- | --- | --- | --- | --- | --- | --- | --- | --- | --- | --- | --- | --- | --- |
|  | **The number of clusters (*k*)** | | | | | | | | | | | | | | |
| **Feature extraction method** | **2** | **3** | **4** | **5** | **6** | | **7** | | **8** | | | **9** | | **10** | |
| Raw data | 78.1 | 63.6 | 54.0 | 47.8 | 42.9 | | 38.9 | | 36.3 | | | 33.3 | | 30.9 | |
| Principal component analysis | 582.0 | 548.0 | 515.0 | 487.9 | 461.2 | | 437.7 | | 413.0 | | | 395.8 | | 385.1 | |
| Deep autoencoder (*J* = 1) | 2566.9 | 3664.3 | 4510.1 | 5803.4 | 6764.1 | | 7634.3 | | 8658.1 | | | 9565.0 | | 10518.6 | |
| Deep autoencoder (*J* = 2) | 3220.0 | 4154.6 | 5189.2 | 6188.3 | 7015.4 | | 7682.8 | | 8453.5 | | | 8993.6 | | 9407.5 | |
| Deep autoencoder (*J* = 3) | 2697.8 | 2926.5 | 3458.9 | 3710.7 | 4092.6 | | 4304.7 | | 4462.9 | | | 4659.4 | | 4647.5 | |
| Deep autoencoder (*J* = 4) | 1976.2 | 2164.0 | 2125.4 | 1979.9 | 1876.4 | | 1887.6 | | 1820.6 | | | 1779.0 | | 1746.1 | |
| Deep autoencoder (*J* = 5) | 2294.4 | 2505.5 | 2537.0 | 2451.6 | 2358.7 | | 2186.8 | | 2076.1 | | | 1995.3 | | 1936.8 | |
| Deep autoencoder (*J* = 6) | 2342.9 | 2299.4 | 2176.5 | 2039.0 | 1864.5 | | 1714.0 | | 1607.6 | | | 1537.0 | | 1474.0 | |
| Deep autoencoder (*J* = 7) | 2145.6 | 2165.9 | 2062.1 | 1948.1 | 1805.1 | | 1659.0 | | 1538.2 | | | 1451.3 | | 1372.7 | |
| Deep autoencoder (*J* = 8) | 2022.9 | 1901.5 | 1752.8 | 1596.3 | 1430.0 | | 1302.6 | | 1207.1 | | | 1132.4 | | 1066.5 | |
| Deep autoencoder (*J* = 9) | 2099.1 | 2007.6 | 1882.7 | 1732.4 | 1569.3 | | 1408.6 | | 1286.2 | | | 1200.6 | | 1131.5 | |
| Deep autoencoder (*J* = 10) | 1953.4 | 1739.6 | 1595.0 | 1447.4 | 1279.1 | | 1138.0 | | 1036.3 | | | 960.9 | | 897.2 | |
| *J* is the number of nodes in the second hidden layer. | | | | | | | | | | | | | | | |

| **Supplementary Table 2. Silhouette coefficient** | | | | | |  | |  | |  |  | |  | |  |
| --- | --- | --- | --- | --- | --- | --- | --- | --- | --- | --- | --- | --- | --- | --- | --- |
|  | **The number of clusters (*k*)** | | | | | | | | | | | | | | |
| **Feature extraction method** | **2** | **3** | **4** | **5** | **6** | | **7** | | **8** | | | **9** | | **10** | |
| Raw data | 0.090 | 0.028 | 0.010 | 0.005 | 0.006 | | 0.002 | | 0.005 | | | -0.005 | | -0.008 | |
| Principal component analysis | 0.277 | 0.202 | 0.187 | 0.186 | 0.189 | | 0.187 | | 0.185 | | | 0.177 | | 0.176 | |
| Deep autoencoder (*J* = 1) | 0.628 | 0.574 | 0.568 | 0.586 | 0.579 | | 0.564 | | 0.568 | | | 0.552 | | 0.552 | |
| Deep autoencoder (*J* = 2) | 0.704 | 0.614 | 0.586 | 0.560 | 0.524 | | 0.523 | | 0.504 | | | 0.492 | | 0.472 | |
| Deep autoencoder (*J* = 3) | 0.620 | 0.527 | 0.497 | 0.473 | 0.455 | | 0.423 | | 0.391 | | | 0.386 | | 0.352 | |
| Deep autoencoder (*J* = 4) | 0.550 | 0.463 | 0.390 | 0.371 | 0.326 | | 0.322 | | 0.294 | | | 0.292 | | 0.281 | |
| Deep autoencoder (*J* = 5) | 0.538 | 0.477 | 0.405 | 0.344 | 0.316 | | 0.302 | | 0.295 | | | 0.268 | | 0.279 | |
| Deep autoencoder (*J* = 6) | 0.524 | 0.412 | 0.348 | 0.312 | 0.259 | | 0.234 | | 0.236 | | | 0.231 | | 0.241 | |
| Deep autoencoder (*J* = 7) | 0.532 | 0.436 | 0.366 | 0.296 | 0.258 | | 0.255 | | 0.239 | | | 0.227 | | 0.217 | |
| Deep autoencoder (*J* = 8) | 0.487 | 0.371 | 0.307 | 0.277 | 0.215 | | 0.211 | | 0.197 | | | 0.197 | | 0.180 | |
| Deep autoencoder (*J* = 9) | 0.503 | 0.370 | 0.339 | 0.267 | 0.243 | | 0.194 | | 0.226 | | | 0.199 | | 0.170 | |
| Deep autoencoder (*J* = 10) | 0.475 | 0.347 | 0.291 | 0.249 | 0.200 | | 0.191 | | 0.177 | | | 0.165 | | 0.150 | |
| *J* is the number of nodes in the second hidden layer. | | | | | | | | | | | | | | | |
